# High myosin binding protein H expression predicts poor prognosis in glioma patients

**DOI:** 10.1038/s41598-022-05512-4

**Published:** 2022-01-27

**Authors:** Jianfei Zhang, Qianqiao Guo, Guoxiang Zhang, Xuemei Cao, Wei Chen, Yong Li, Minwu Guan, Jianjun Yu, Xindong Wang, Yujin Yan

**Affiliations:** 1 Department of Neurosurgery, The Affiliated Hospital of Medical School of Ningbo University, Ningbo, 315020 China; 2 Department of Electrophysiology, The Affiliated Hospital of Medical School of Ningbo University, Ningbo, 315020 China; 3 Department of General Surgery, Lianshi People’s Hospital, Nanxun District, Huzhou, 313013 China; 4 Ningbo Clinical Pathology Diagnostic Center, Ningbo, 315020 China; 5 Zhejiang Key Laboratory of Pathophysiology, Ningbo University, Ningbo, 315211 China

**Keywords:** CNS cancer, Tumour biomarkers

## Abstract

Glioma is the most common and fatal primary brain tumor in humans. Myosin binding protein H (MYBPH), which was first identified as an important myofibrillar constituent of vertebrate skeletal and cardiac muscles, reduces cell motility and metastasis. However, its role in gliomas remains unclear. We evaluated the expression of MYBPH in glioma using Gene Expression Profiling Interactive Analysis (http://gepia.cancer-pku.cn/) and Chinese Glioma Genome Atlas (https://www.cgga.org.cn/). The results showed that MYBPH was highly expressed in glioma tissues. Moreover, MYBPH expression was significantly associated with high tumor aggressiveness and poor outcomes in glioma patients. Mechanistically, the results suggested that MYBPH might promote tumor progression by improving tumor invasion and migration. Our results establish MYBPH as an important prognostic biomarker that could be considered a potential epigenetic and immunotherapeutic target for treatment. We showed that MYBPH is a novel biomarker that is variably expressed in glioblastoma (GBM). The association of high MYBPH expression with poor prognosis in newly diagnosed GBM patients and increased expression in recurrent GBM is indicative of its role in tumor aggressiveness.

## Introduction

Glioma is the most common primary malignant tumor of the central nervous system^1^. According to the World Health Organization (WHO) classification, gliomas are traditionally classified as low-grade gliomas (LGGs) (WHO I, pilocytic astrocytoma; WHO II, diffuse astrocytoma) and high-grade gliomas (HGGs) (WHO III, anaplastic astrocytoma; and WHO IV, glioblastoma). Despite the emerging advances in diagnostic and therapeutic techniques, the prognosis of malignant glioma patients remains unsatisfactory^2,3^. Therefore, there is an urgent need for further development in clinical diagnosis and treatment techniques for glioma, for discovering new molecular markers and prognostic factors, and for exploring new therapeutic targets that can improve the prognosis and quality of life of patients with glioma.

Glioblastoma (GBM) is the most prevalent and malignant primary brain tumor in adults, with a 5-year survival rate of less than 5% and a median survival duration of 9–12 months^4^. One of the most significant clinical features of GBM is its invasiveness. Although GBM rarely metastasizes to extracranial sites, its ability to be widely invasive in the brain contributes to its highly malignant behavior^5^. Cell motility is one of the most important aspects of tumor invasion and it is regulated by a variety of growth factor receptors that are imbalanced or amplified in GBM^6^. Although these receptors can be targeted with specific drugs, the effectiveness of drug treatment is weakened to a great extent by signal cascade redundancy in GBM^7,8^. Therefore, more effective targets for tumor cell invasion are required.

GBM invasion is driven and controlled by the cytoskeletal network of cells. For example, myosin II plays an important role in the progression of malignancy in glioma, which is beneficial for intercellular communication and invasion^9^. However, the expression pattern and clinical characteristics of myosin binding protein H (MYBPH) in GBM have not yet been described. MYBPH is an important myofibril component of vertebrate skeletal muscles and myocardium^10^. Research suggests that MYBPH is a transcriptional target of thyroid transcription factor-1 (TTF-1), which reduces cell movement and metastasis by inhibiting Rho-associated protein kinase 1 (ROCK1)^11^. Research has shown that ROCK is closely related to nuclear squeezing and glioblastoma cell invasion^12^.

In the present study, we investigated the expression levels of MYBPH and also evaluated the association between MYBPH expression levels and prognosis of glioma. Using immunohistochemistry, we confirmed that MYBPH expression is upregulated in GBM. Furthermore, we used publicly available datasets to confirm the association between high MYBPH mRNA levels and poor outcomes in GBM. Furthermore, we explored the role of MYBPH in the biological behavior of a glioma cell line.

## Results

### MYBPH expression is upregulated in GBM tissues

**Figure 1:**
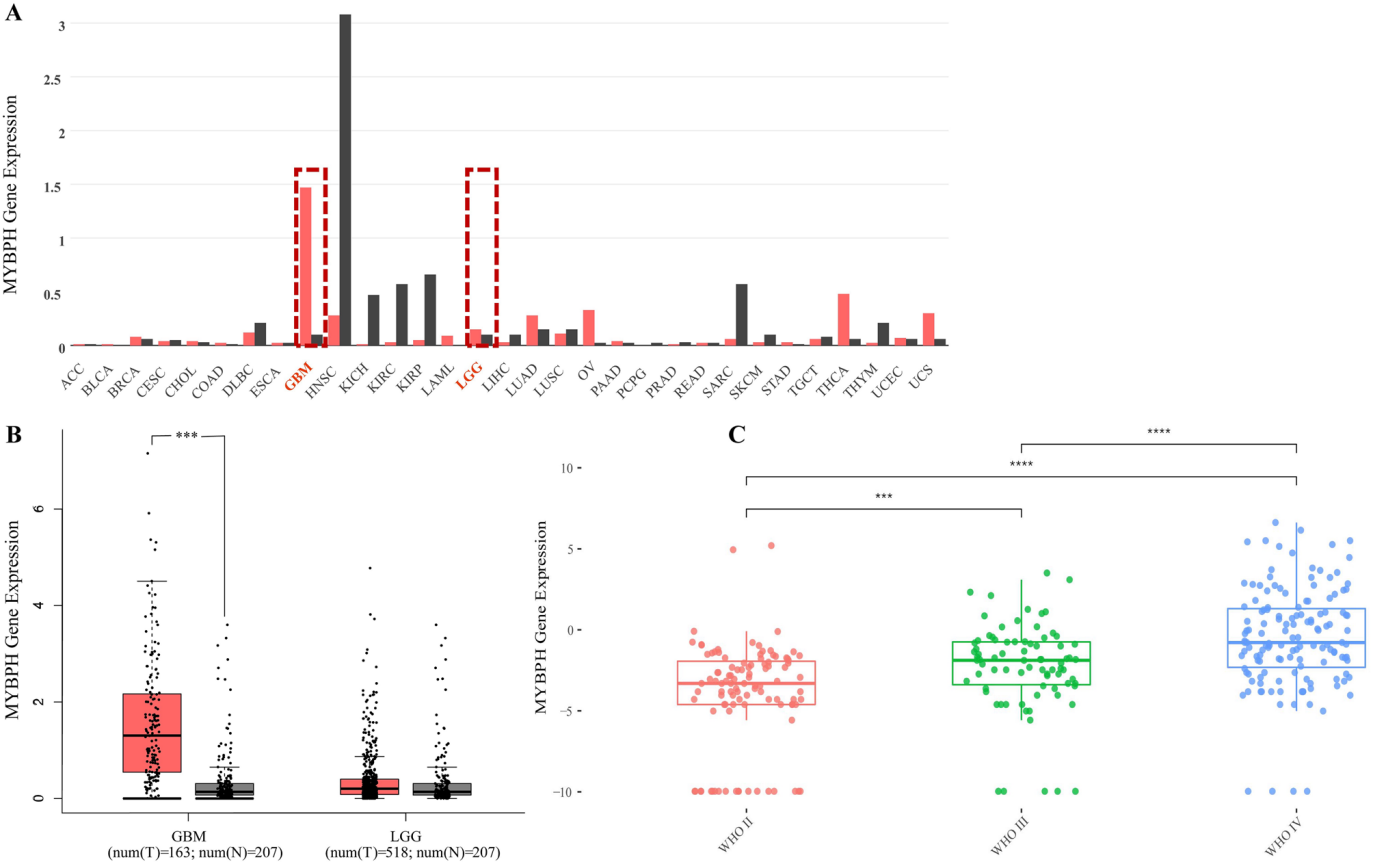
The public database-based expression of MYBPH in glioma tissues. The gene expression profile across 31 tumor samples and paired normal tissues from TCGA data. Among them, the red dashed boxes showed that the expression of MYBPH in human glioma (**A**). The expression of MYBPH messenger RNA (mRNA) was detected from the GEPIA and CGGA public databases (**B**, **C**).

**Figure 2:**
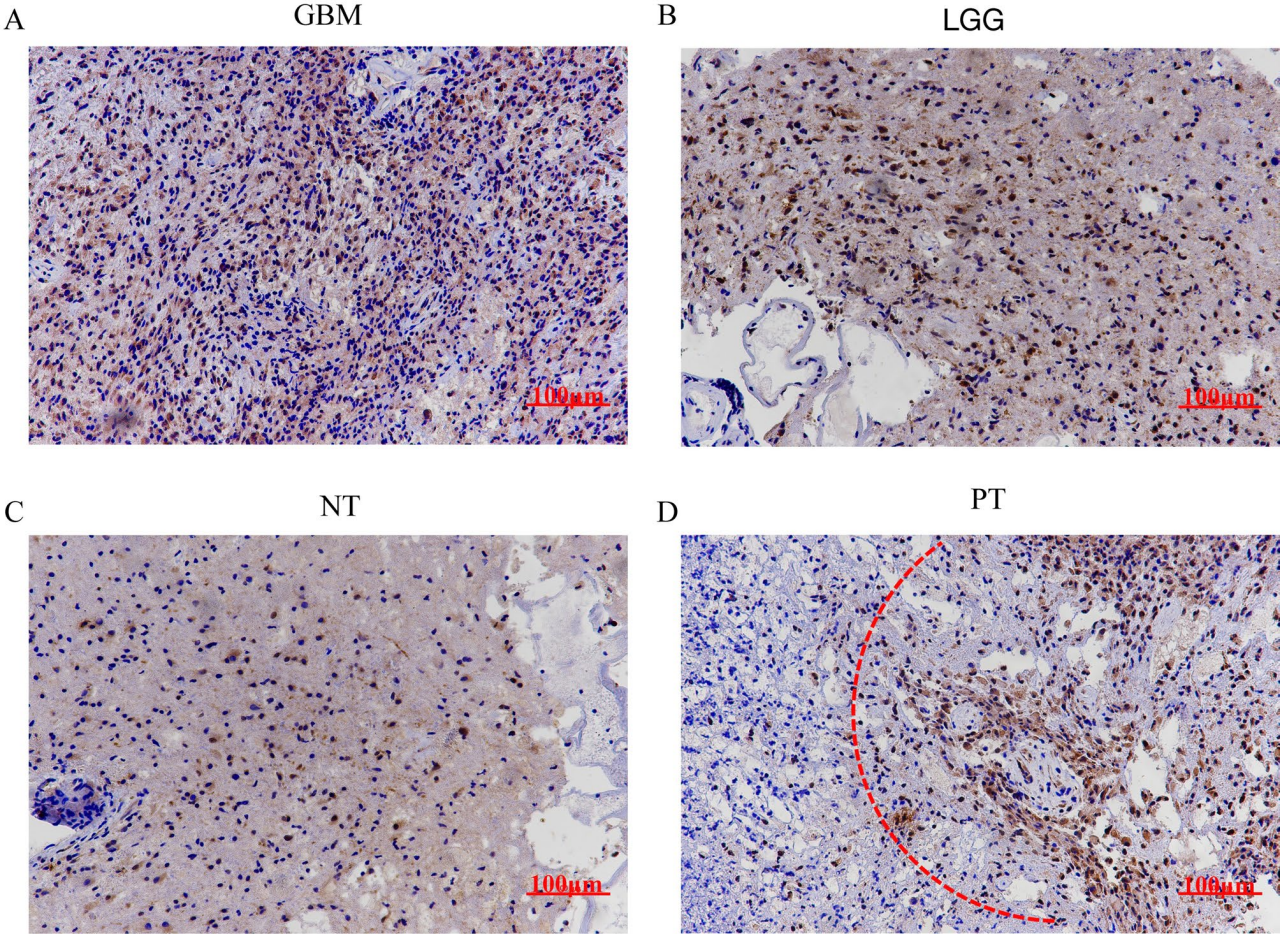
MYBPH expression in human glioma tissues, peritumor tissues and normal tissues. (**A**, **B**) Positive MYBPH immunostaining could be found in glioma tissues (**A** strong, **B** weak). (**C**, **D**) Weak staining of MYBPH immunostaining could be found in normal tissues and peritumor tissues. The red dashes denote the dividing line between normal tissue and tumor tissue. GBM: glioblastoma; LGG: low-grade glioma; NT: normal tissue; PT: peritumor tissue. Scale bar: 100 μm.

Using online databases, we found that MYBPH was overexpressed in GBM tissues (Fig. 1). In addition, immunohistochemical results showed that MYBPH was upregulated in clinical specimens from GBM patients and MYBPH expression was higher in tumor tissues than in the corresponding peritumor tissues and normal tissues (Fig. 2). MYBPH expression was positively associated with glioma grade (*P* = 0.002) and KPS score (*P* = 0.022). There were no significant relationships between MYBPH expression and other clinicopathological features of gliomas, including the patients’ gender, age, and tumor size (all *P* values > 0.05; Table 1). Therefore, MYBPH expression is expected to be positively correlated with glioma grade, hinting that it might have a functional role.

**Table 1:** Association of MYBPH expression with clinical parameters of glioma patients.

Parameter	No. of patients	MYBPH expression,		*P* value
		Low (n, %)	High (n, %)	
**WHO grade**				
I	7	6(85.71)	1(14.19)	0.002
II	8	5(62.50)	3(37.50)	
III	11	4(36.36)	7(63.64)	
IV	14	2(14.29)	12(85.71)	
**Gender**				
Male	21	10(47.62)	11(52.38)	0.987
Female	19	9(47.37)	10(52.63)	
**Age (years)**				
≤ 50	23	10(43.48)	13(56.52)	0.822
> 50	17	8(47.06)	9(52.94)	
**Tumor size (cm)**				
≤ 5	25	12(48.00)	13(52.00)	0.935
> 5	15	7(46.67)	8(53.33)	
**KPS score**				
≤ 90	28	6(21.43)	22(78.57)	0.022
> 90	12	7(22.22)	5(77.78)	

### Analysis of MYBPH expression with 1p/19q codel status and isocitrate dehydrogenase (IDH) mutation status

**Figure 3:**
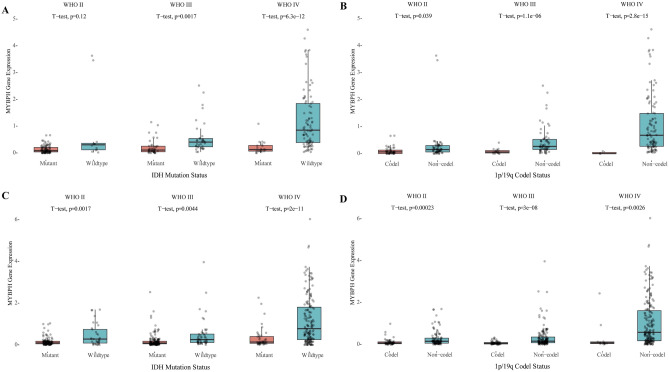
Expression of MYBPH in the CGGA database based on the tumor grade with IDH mutation status and 1p/19q codel status. (**A**) IDH status-stratified distribution of MYBPH expression (Dataset ID: mRNAseq_325). (**B**) 1p19q status stratified by the distribution of MYBPH expression (Dataset ID: mRNAseq_325). (**C**) IDH status stratified by the distribution of MYBPH expression (DataSet ID: mRNAseq_693). (**D**) MYBPH expression distribution stratified by 1p19q status (Dataset ID: mRNAseq_693).

**Figure 4:**
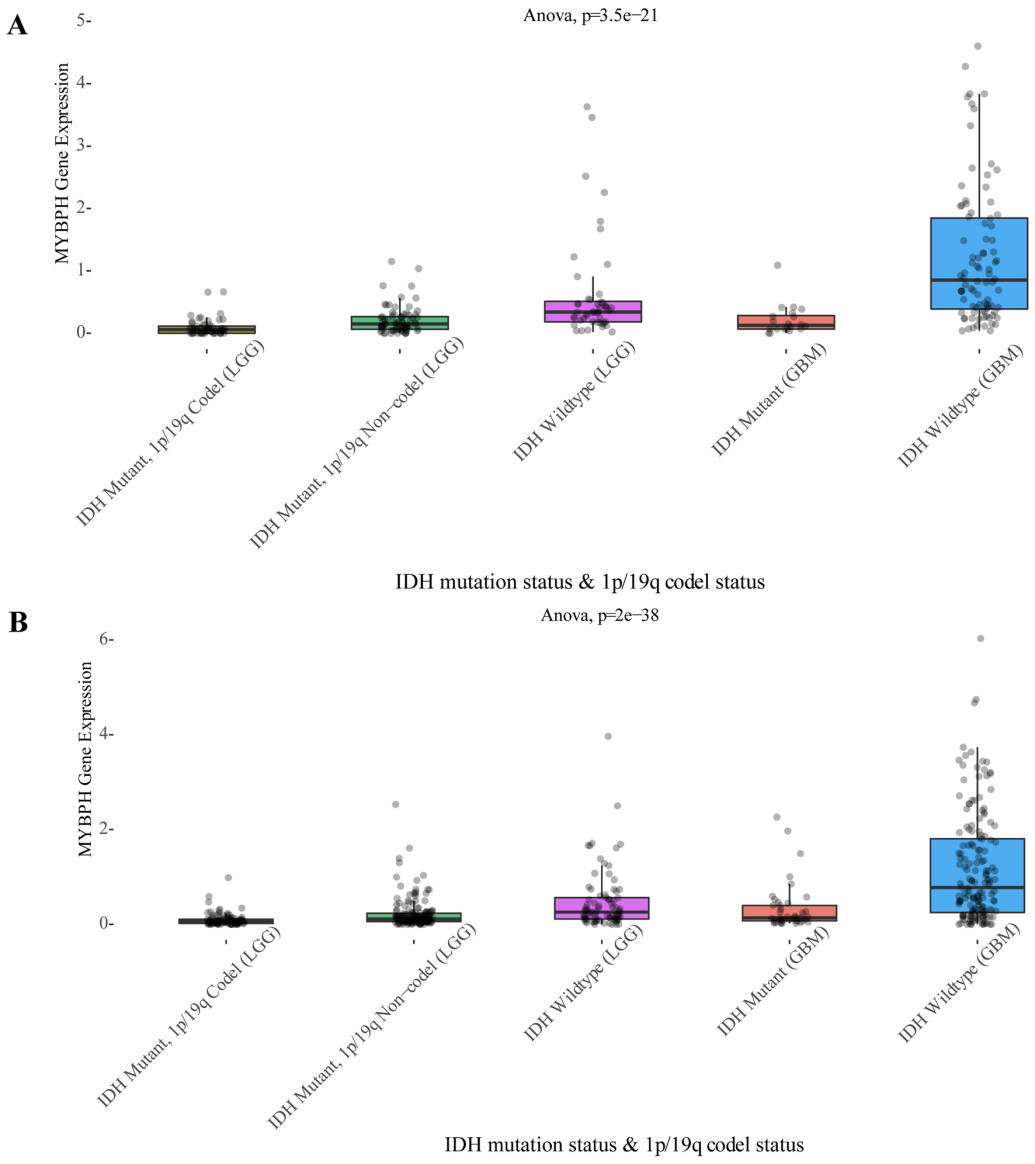
Expression of MYBPH based on the IDH mutation status and 1p/19q codel status. (**A**) MYBPH expression distribution stratifified by 1p19q status and IDH status (Dataset ID: mRNAseq_325). (**B**) MYBPH expression distribution stratifified by 1p19q status and IDH status (Dataset ID: mRNAseq_693).

We used two different datasets from the CGGA database and analysed MYBPH expression based on the classic WHO grades with respect to 1p/19q codel status and IDH mutation status. In the IDH-wildtype and 1p/19q non-codel groups, MYBPH expression showed a trend for increase from LGG (WHO II) to HGG (WHO IV) in the datasets (ID: mRNAseq_325 and ID: mRNAseq_693) (Fig. 3). Interestingly, the lowest level of MYBPH expression was observed in the IDH-mutant and 1p/19q codel groups (LGG) and the highest level of expression was observed in the IDH-wildtype group (GBM) (Fig. 4). Generally, these results suggest that MYBPH expression was significantly increased in HGG, especially in GBM, and indicated a poor prognosis.

### MYBPH expression is related to the prognosis of glioma patients

**Figure 5:**
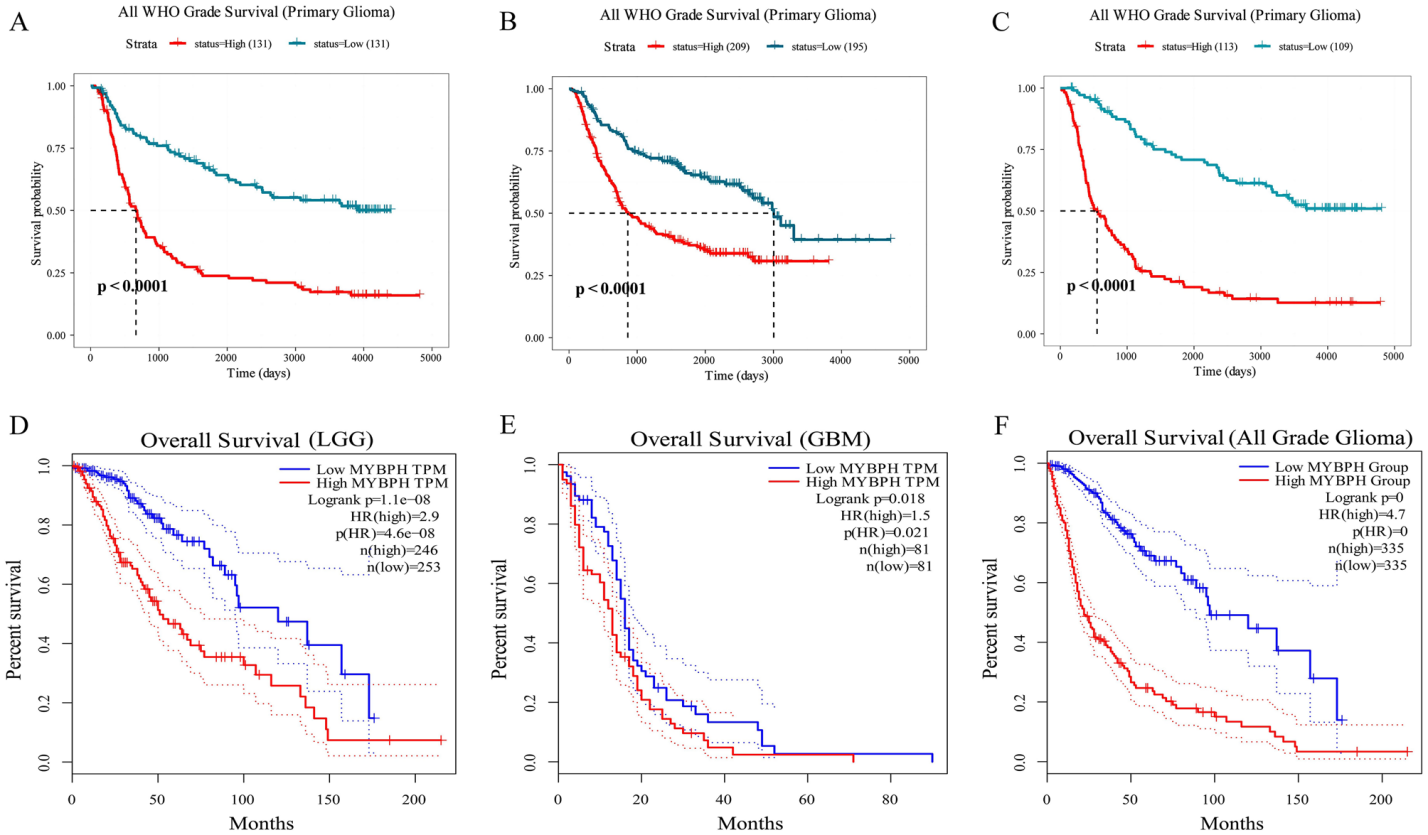
Kaplan–Meier survival curve analysis of overall survival (OS) of glioma patients according to MYBPH expression. (**A**), (**B**) and (**C**) showing K-M survival curves for overall survival for MYBPH in the CGGA (Dataset ID: mRNAseq_301, Dataset ID: mRNAseq_693 and Dataset ID: mRNAseq_325). (**D**), (**E**) and (**F**) are the K-M survival curves for MYBPH in the GEPIA.

To further illustrate the relationship between survival and MYBPH expression in glioma, we analysed two public databases, namely the GEPIA database and three CGGA datasets. The group with higher MYBPH expression had a poor prognosis of primary glioma in Dataset 1 (ID: mRNAseq_325) (*P* < 0.0001, Fig. 5A). Similar results were observed in Dataset 2 (ID: mRNAseq_693) and in Dataset 3 (ID: mRNAseq_301) (*P* < 0.0001, Fig. 5B,C). Analysis of the GEPIA database revealed that high levels of MYBPH were associated with poor prognosis in both LGG and GBM (*P* < 0.05, Fig. 5D–F). These results indicated that MYBPH might be used as a potential biomarker for the prognostic evaluation of gliomas.

### Knockdown of MYBPH impaired migration of glioma cells

**Figure 6:**
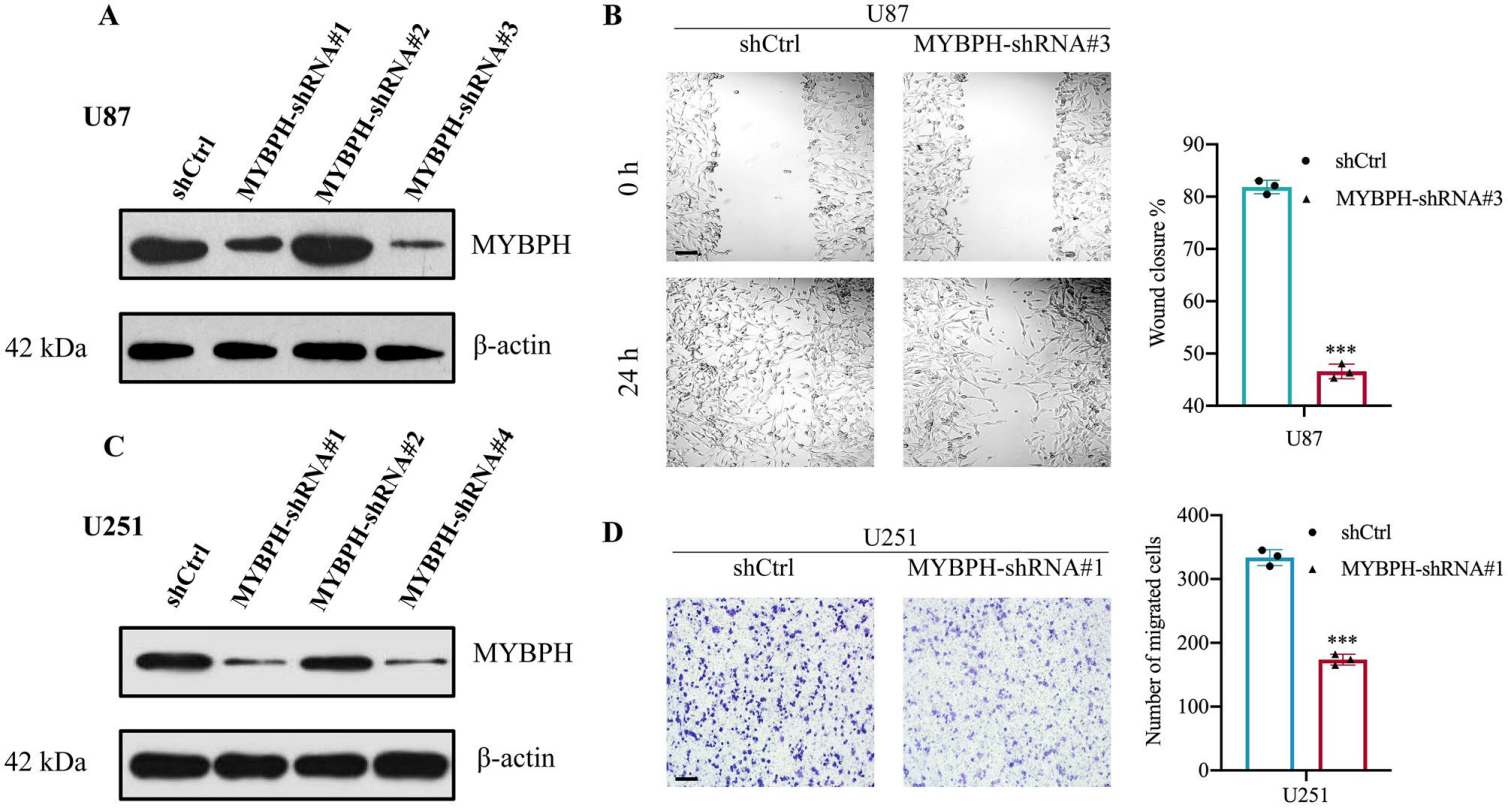
Effect of knockdown of MYBPH on cell viability in glioma cell lines using wound healing assay and transwell migration assay. (**A**, **C**) U251 and U87 glioma cells were transfected with shMYBPH to stably reduce MYBPH expression. (**B**) The wound healing assay showed that knockdown of MYBPH significantly attenuated the cell migration ability in U87 cells at 24 h after scraping. (Scale bar, 100 μm.) (**D**) The number of crystal violet-stained cells was significantly decreased in the MYBPH-shRNA group compared with the MYBPH-NC group in U251 cells, magnification, Scale bar = 100 μm in 200 magnification. Data are shown as the mean ± SEM; n = 3, (***P < 0.001).

To explore the possible contribution of MYBPH to the progression of GBM, effect of MYBPH knockdown on cell migration was examined by wound healing and transwell assays. To do this, we knocked down MYBPH in glioma cells using shRNA constructs. In addition, we checked three shRNAs of MYBPH individually and selected the best silencing effect of shMYBPH for subsequent functional studies (Fig. 6A,C). Downregulation of MYBPH attenuated the migration capacity of glioma cells in the wound healing and transwell assays (Fig. 6B,D).

**Figure 7:**
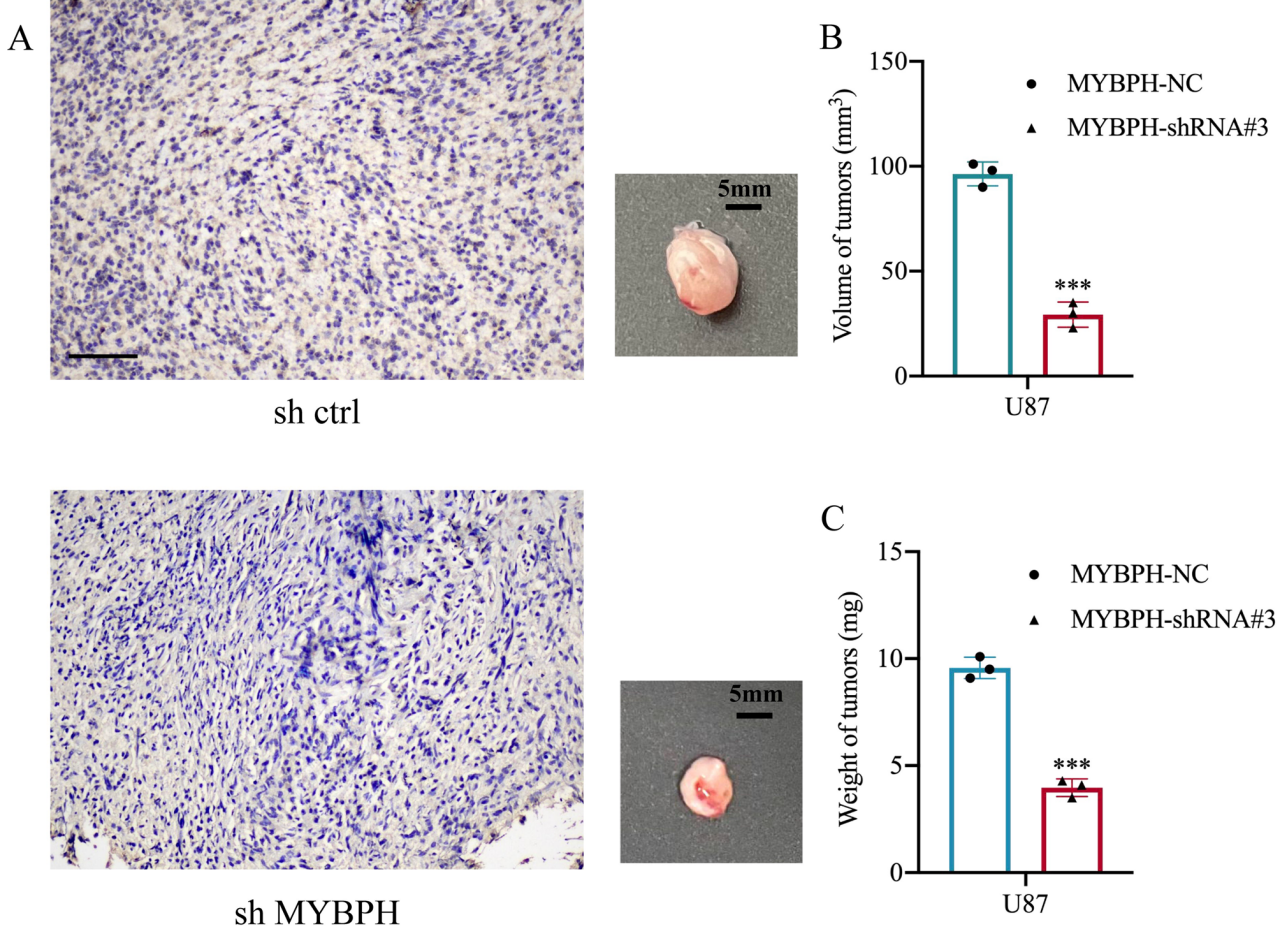
Effect of MYBPH on glioma tumorigenesis. (**A**) U87 cells (5 × 10^6^/100 μl) were injected subcutaneously into the left upper back of NOD-SCID mice, and after 10 days, mice were sacrificed. Representative samples showing the results of immunohistochemical analysis, performed using the anti-MYBPH antibody. (**B**, **C**) The volume and weight of tumors were significantly decreased in the MYBPH-shRNA group compared with the MYBPH-NC group in U87 cells. Data are shown as mean ± SEM; n = 3, ***P < 0.001. Scale bar: 100 μm.

In order to further investigate the influence of MYBPH knockdown on tumor growth in vivo, we constructed mice xenograft models via the subcutaneous injection of U87 cells transfected with either shMYBPH (shMYBPH#3) or shCtrl. Then we measured tumor weight and tumor volume, experimental results showed that the tumor growth rate of the shMYBPH group was slower than that of the shCtrl group. The same results were observed in the volume of tumors (*P* < 0.001, Fig. 7 and Figure S5). Animal experiments indicated that knockdown of MYBPH reduced tumorigenicity in vivo. These findings indicate that MYBPH might play an important role in glioma progression. To avoid off target effects, we rescued 1 shRNA with overexpression of MYBPH and conducted relevant experiments. For that these results are presented in supplementary Information file (Figure S1).

Furthermore, we also performed CCK-8 assay, and flow cytometry analysis of the cell apoptosis and the cell cycle assay. Knockdown of MYBPH in glioma cells could not affect the cell viability, apoptosis and cell cycle (Supplementary Fig. S2-4). These experimental results showed that MYBPH contributes to the progression of glioma by promoting cell migration. In fact, as an important myofibril component, MYBPH mainly affects cell movement. Some studies have shown that MYBPH can reduce cell movement and metastasis by inhibiting ROCK1^11^ and inhibit NM IIA assembly to reduce cell motility by directly interacting with NMHC IIA.

## Discussion

It is well known that malignant glioma, the deadliest type of brain cancer, is characterized by high proliferation, invasion, and neurological destruction^13^. Diffuse invasive growth of tumor cells is a critical challenge in the clinical management of glioma patients. Therefore, it is essential to identify prognostic markers and therapeutic targets.

In the present study, we aimed to investigate a novel biomarker, MYBPH, for its clinical utility in GBM. We observed that MYBPH expression was higher in GBM tissues than in corresponding peritumor tissues and normal tissues and its expression was significantly associated with glioma grade (*P* = 0.002) and a lower Karnofsky Performance Scale score (*P* = 0.022). However, the correlations with patients’ sex, age, and tumor size were not significant (*P* > 0.05). The ‘survival’ modules of GEPIA and CGGA were used to test the prognostic value of MYBPH in glioma patients.

Further analysis revealed a correlation between MYBPH expression and IDH1 expression. IDH1 phenotypes have been strongly recommended as a new diagnostic method for clinical applications^14^. The present study confirmed a significant difference between 1p/19q codel status and non-codel status as well as between the IDH1-mutant group and the wild-type group. Moreover, poor survival in the IDH1- mutant group was associated with higher expression of MYBPH. These results suggest that MYBPH could be a prognostic marker in the evaluation of glioma patients.

Fundamentally, the molecular mechanism of cell movement is closely related to cell survival, which in turn may be related to metastasis^15^. However, cell migration is not a single phenomenon. Under completely different physiological backgrounds, different cell types show different morphologies, cell–cell interactions, and types of movement. Cell migration behavior is related to many forces such as actomyosin contractility and actin polymerization-mediated protrusion^16,17^. In fact, cell migration driven by myosin filament assembly is important for tumor invasion. MYBPH has been reported to be a transcriptional target of TTF-1 and inhibits ROCK1 activity to reduce cell motility and metastasis in lung adenocarcinoma cells^11^. Furthermore, MYBPH can also inhibit NM IIA assembly to reduce cell motility by directly interacting with NMHC IIA. In a rat carotid balloon injury model, MYBPH could inhibit vascular smooth muscle cell migration and attenuate neointimal hyperplasia by inhibiting ROCK1^18^. Recent studies have suggested that MYBPH can be used to predict the prognosis of invasive breast cancer and lung adenocarcinoma^19,20^. In the present study, we showed that knockdown of MYBPH attenuated cell migration and reduced tumorigenicity in vivo.

In conclusion, our findings indicate that MYBPH is positively correlated with the prognosis and grade of glioma. Several experimental studies on cells and animals have demonstrated that MYBPH might play an important role in tumor progression. The present study has some limitations including a lack of in-depth study of the mechanism. Future studies need to expand the sample size to validate the results of the present study. In conclusion, MYBPH might serve as a valuable prognostic marker and a potential therapeutic target for glioma.

## Materials and methods

### Ethical statement and informed consent

This study was approved by the Ethics Review Committee of the Affiliated Hospital of the School of Medicine, Ningbo University (permission no.: NBU-2020–039). All procedures were in accordance with the *ARRIVE guidelines* and the animal experiment obeyed with the *Declaration of Helsinki*.

### Tissue samples

Glioma tissue sections were obtained from the Department of Neurosurgery, Affiliated Hospital of School of Medicine, Ningbo University (Ningbo, Zhejiang, China). According to the WHO classification criteria, all tumor tissue samples were divided into the following two groups: low-grade (grades I–II, 15 cases) and high-grade (grades III–IV, 25 cases). Additionally, 10 tumor-adjacent brain tissue samples and eight normal brain tissue samples were included. Table 1 presents the detailed clinical parameters of the patients.

### Analysis of expression and survival

Using the online database analysis tool, we explored MYBPH expression in glioma tissues and in normal tissues. In addition, two databases, namely Gene Expression Profiling Interactive Analysis (GEPIA) and Chinese Glioma Genome Atlas (CGGA) were used to explore the expression of MYBPH in glioma tissues. Using the ‘survival’ modules of GEPIA and CGGA, we evaluated the correlation between MYBPH expression and prognosis of glioma.

### Cell culture, reagents, and antibodies

U251 and U87 cell lines were obtained from the Chinese Academy of Sciences (Shanghai, China). Cells were cultured in Dulbecco’s modified Eagle’s medium (DMEM) supplemented with 10% fetal bovine serum (FBS) (HyClone, Logan, UT, USA) and 100 IU/ml penicillin–streptomycin in a cell incubator with 5% CO_2_ at 37 °C. The MYBPH antibody (PA5-44,583) was purchased from Invitrogen (Carlsbad, CA, USA).

### Lentivirus construction establishment and transfection

MYBPH (accession number: NM_004997) was knocked down by lentivirus-mediated transfection and implemented with short hairpin (sh) RNAs or scrambled controls in pLKO.1-puro vector (5′–3′, shMYBPH#1: CTACAGTCAAACTCCAGAGAT; shMYBPH#2: GCCAAACCTAAA.

GGGTTTATT; shMYBPH#3: CTACACCTGCAAGGCCATAAA). The shRNA vectors were purchased from Sigma-Aldrich. And at the meantime, the CDS sequence of MYBPH was sub-cloned into the BamH1, XhoI, and Notl sites of pCDH1-CMVBXN-EF1-Neo vector (Invivogen, San Diego, CA) to generate the pCDH1-MYBPH plasmid (recombinant expression vector of MYBPH), and packaged recombined lentivirus. Briefly, glioma cells transfection was conducted in accordance with the manufacturer’s protocol of Lipofectamine 2000 (Invitrogen, CA, United States).

### Wound-healing migration assay

Briefly, cells were cultured in 6-well plates (Corning, San Diego, CA, USA) at a density of 1 × 10^5^ cells/ml. A 500 μm wide scratch was created using a 200-μL pipette tip when the cells reached 90% confluence. Cells were washed twice with phosphate-buffered saline (PBS) and incubated in serum-free DMEM at 37 °C for 48 h in a 5% CO_2_ incubator. The migration of cells into the wounded area was recorded at two different time points (0 and 24 h) using an inverted microscope (20 × magnification).

### Transwell migration assay

The experimental procedure of transwell migration assay was performed as previously described^21^. The migration and invasion ability of glioma cells were performed using transwell chambers (Corning Costar 3422, San Diego, CA, USA). Briefly, the filters of the upper wells were coated with Matrigel, and the lower wells were filled with DMEM medium supplemented with 10% FBS as a chemoattractant. Cells were trypsinized and suspended at a density of 1 × 10^6^ cells/ml in DMEM medium containing 10% fetal bovine serum. Subsequently, 100 μL of cell suspension was loaded into collagen-coated transwell chambers (migration) in triplicate. After incubation for 12 h at 37˚C. Non-migrated cells on the upper side were removed with a cotton swab. Then, the lower surface of the transwell were fixed with methanol and stained with crystal violet. The number of cells was counted per field from 5 random fields of each membrane, under an optical microscope. The mean values from three independent experiments performed in duplicate were used. The data were presented as mean ± standard deviation.

### Tumorigenicity assay

Four-week-old female non-obese diabetic mice with severe combined immunodeficiency were obtained from Charles River Laboratories, Inc. (Wilmington, MA, USA), acclimatized for 2 weeks, and maintained in a clean room. Subsequently, the mice were returned to their cages and allowed free access to food and water. They were randomly divided into four groups of eight animals each. U87 cells were trypsinized, washed with PBS, resuspended in PBS, and adjusted to a concentration of 5 × 10^6^/100 μL in PBS. Subsequently, the cell suspensions were injected into the left upper back of mice and allowed to grow until tumor formation occurred. Tumor volume was calculated using the following formula (mm^3^): *V* = *(length* × *width*^2^*)*/2. Animal studies were conducted in accordance with the institutional guidelines for the care and use of laboratory animals.

### Statistical analysis

Data were expressed as mean and range or mean ± standard deviation of three independent experiments. Statistical analysis was conducted using paired Student’s t-test, Wilcoxon signed-rank test, and chi-squared test or log-rank survival analysis where appropriate for the final analysis of the data. All statistical analyses were performed using the GraphPad Prism 5.0 software package (GraphPad Software, Inc.; San Diego, CA, USA). Statistical significance was set at *P* < 0.05.

### Ethical approval

The present study was approved by the local Ethics Committee at The Affiliated Hospital of Medical School of Ningbo University (NBU-2020–039). As this study was retrospective, informed consent was waived by the local Ethics Committee at The Affiliated Hospital of Medical School of Ningbo University. All procedures performed in the present study were in accordance with 1964 Helsinki Declaration and its later amendments.

## Supplementary Information

Supplementary Information.

## Data Availability

All data generated or analysed during this study are included in this published article.
