# Supplementary material for: High myosin binding protein H expression predicts poor prognosis in glioma patients
Source: Sci Rep. 2022 Jan 27;12:1525. doi: 10.1038/s41598-022-05512-4 (PMC8795254; doi:10.1038/s41598-022-05512-4)
Supplement: Supplementary file 1 — Supplementary Information. [file 41598_2022_5512_MOESM1_ESM.pdf]

High Myosin Binding Protein H Expression Predicts Poor Prognosis in Glioma Patients

Jianfei Zhang[1,5#], Qianqiao Guo[2#], Guoxiang Zhang[3#], Xuemei Cao[4], Wei Chen[1], Yong Li[1], Minwu Guan[1], Jianjun Yu[1], Xindong Wang[1], Yujin Yan[1*]

[#]*equal contribution*

[1]*Department of Neurosurgery, The Affiliated Hospital of Medical School of Ningbo University, Ningbo 315020, China*

[2]*Department of Electrophysiology, The Affiliated Hospital of Medical School of Ningbo University, Ningbo 315020, China*

[3]*Department of General Surgery, Lianshi People's Hospital, Nanxun District, Huzhou 313013, China*

[4]*Ningbo Clinical Pathology Diagnostic Center, Ningbo 315020, China*

[5]*Zhejiang Key Laboratory of Pathophysiology, Ningbo University, Ningbo 315211, China*

Correspondence to Yujin Yan: Department of Neurosurgery, The Affiliated Hospital of Medical School of Ningbo University, Ningbo 315211, China. 13736090092@163.com

Supplementary Fig. S1

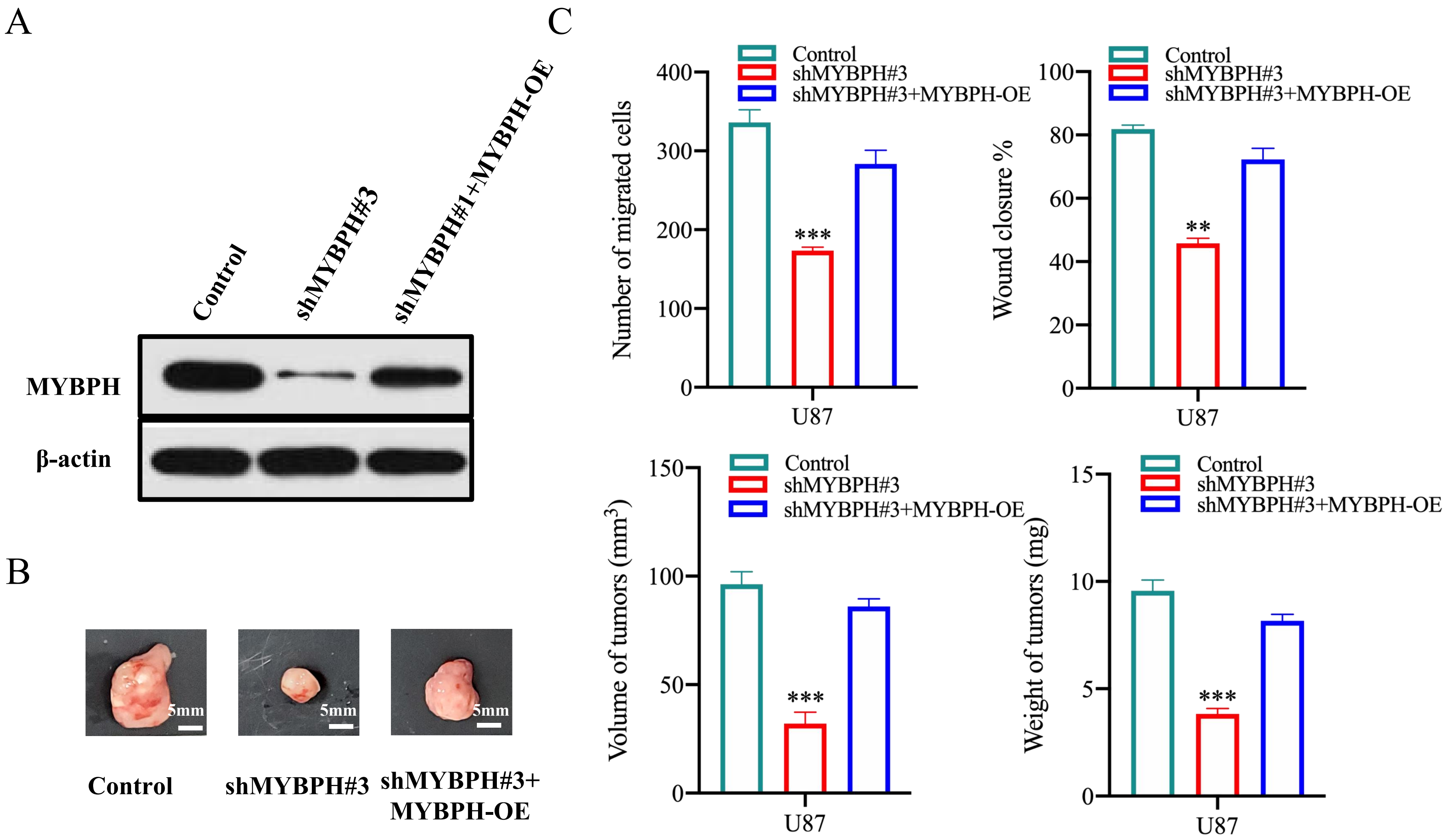

Figure S1. MYBPH overexpression restored the migration capacity of U87 cells and tumor growth *in vivo* caused by MYBPH knockdown. (A) MYBPH protein level was determined by western blot. (B) U87 cells were transfected with either shMYBPH#3, shCtrl or shMYBPH#3+MYBPH-OE. At 48 h after transfection, cells were injected into the left upper back of mice and allowed to grow until tumors formed. Representative samples showing the results of tumorigenicity assay. (C) Data are shown as mean ± SEM; n =3, **p < 0.01, ***p < 0.001.

Abbreviations: shCtrl, Control; shMYBPH#3, MYBPH-shRNA#3; MYBPH-OE, MYBPH overexpression.

Supplementary Fig. S2

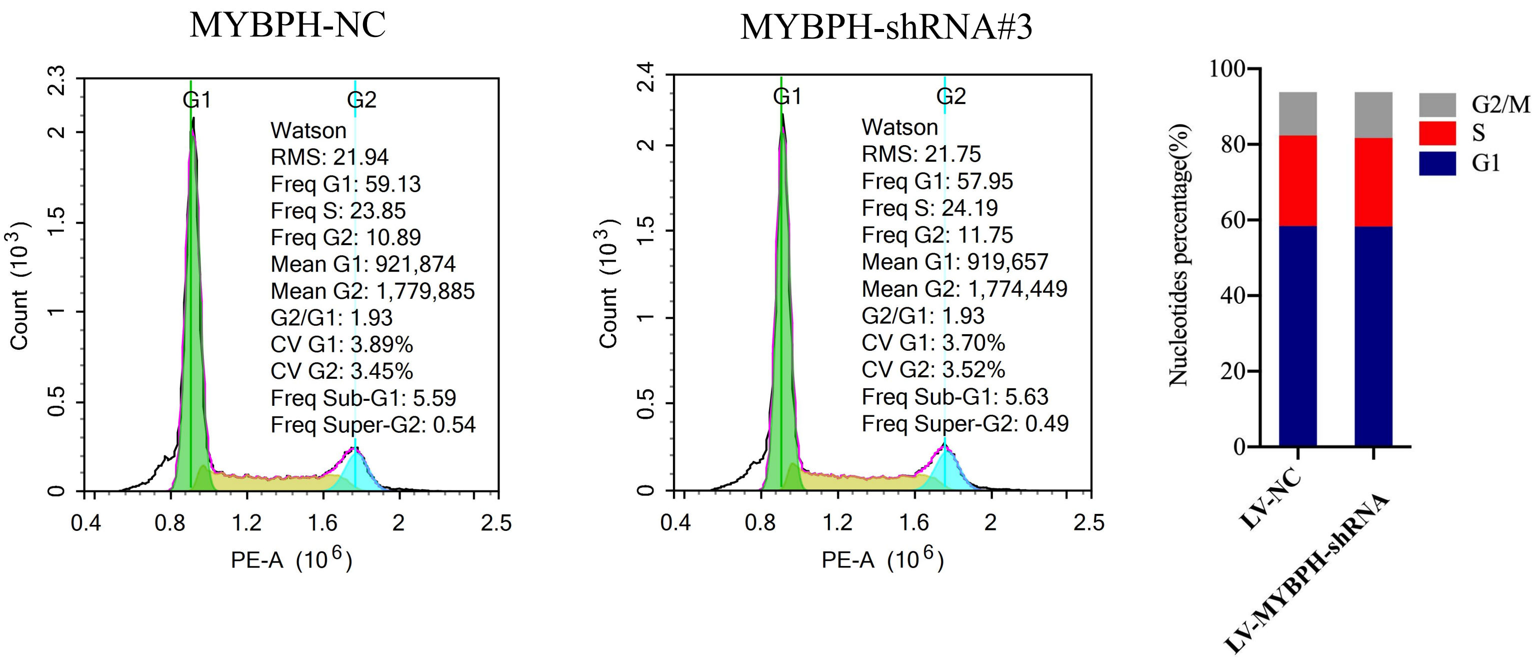

Figure S2. Effect of knockdown of MYBPH on cell cycle in U87 cells. When the MYBPH expression was decreased, the ratio of U87 cells in S, G2/M and G1 phase was not significantly increased in the LV-MYBPH-shRNA group compared with the LV-NC group.

Supplementary Fig. S3

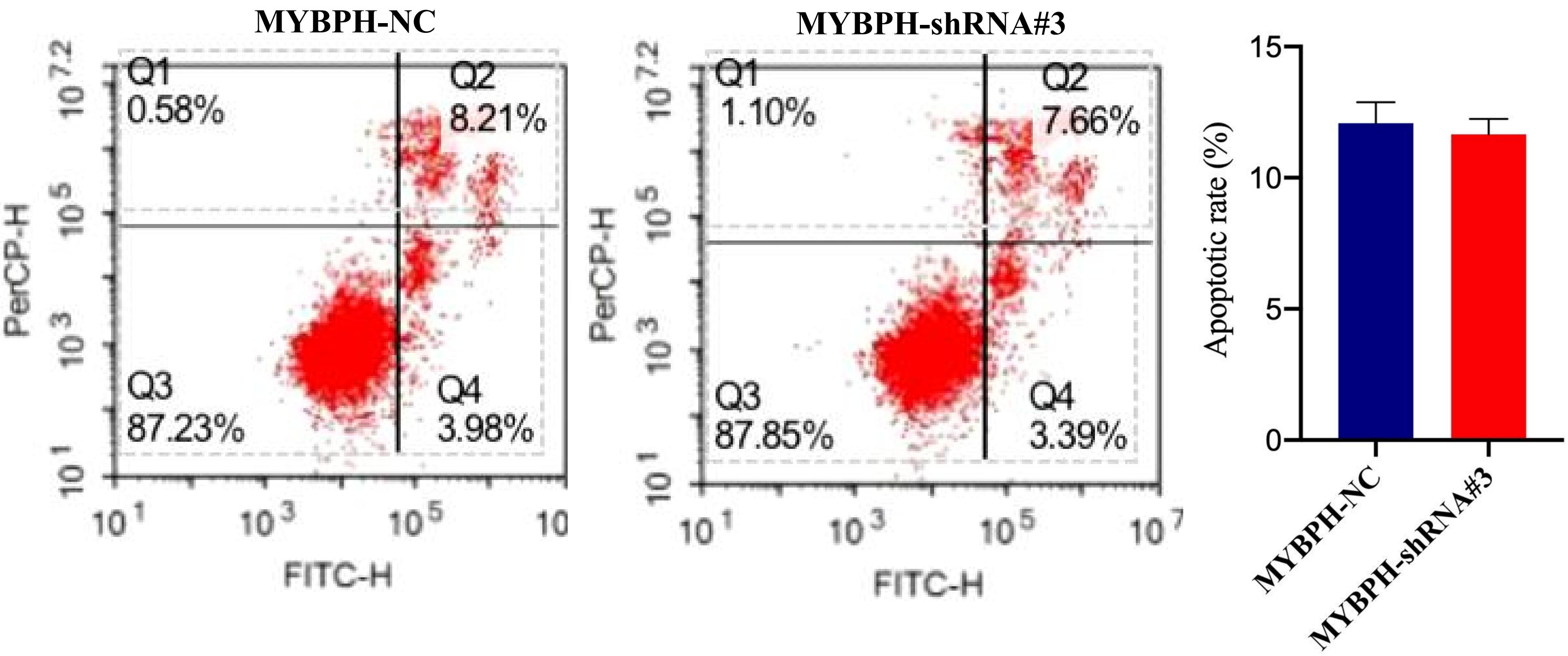

Figure S3. Effect of knockdown of MYBPH on cell apoptosis in U87 cells. Cell apoptosis was measured by flow cytometry. Data were expressed as mean ± SD from three independent experiments.

Supplementary Fig. S4

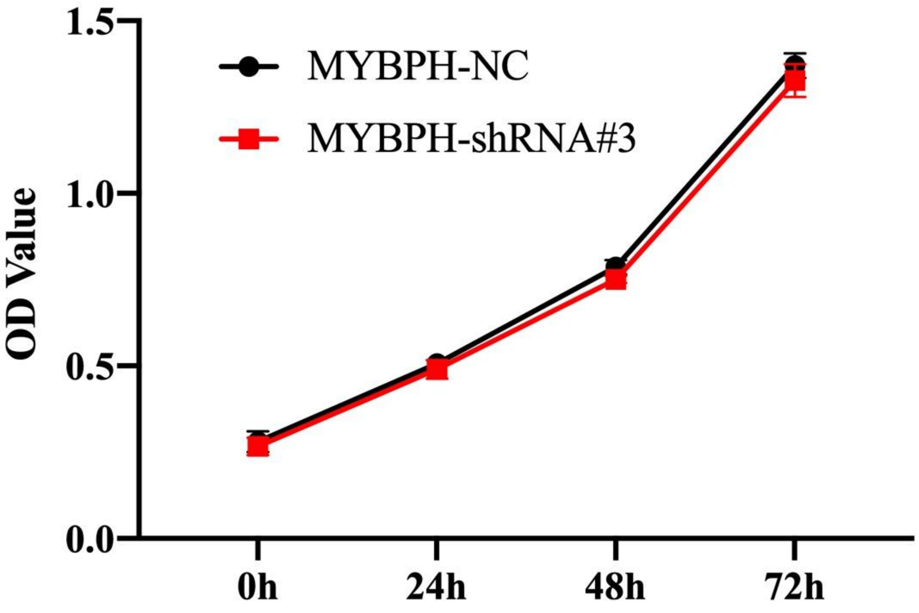

Figure S4. Effect of knockdown of MYBPH on cell proliferation in U87 cells. CCK-8 assay was performed to evaluate the proliferation capacity of U87 cells with different transfections.

Supplementary Fig. S5

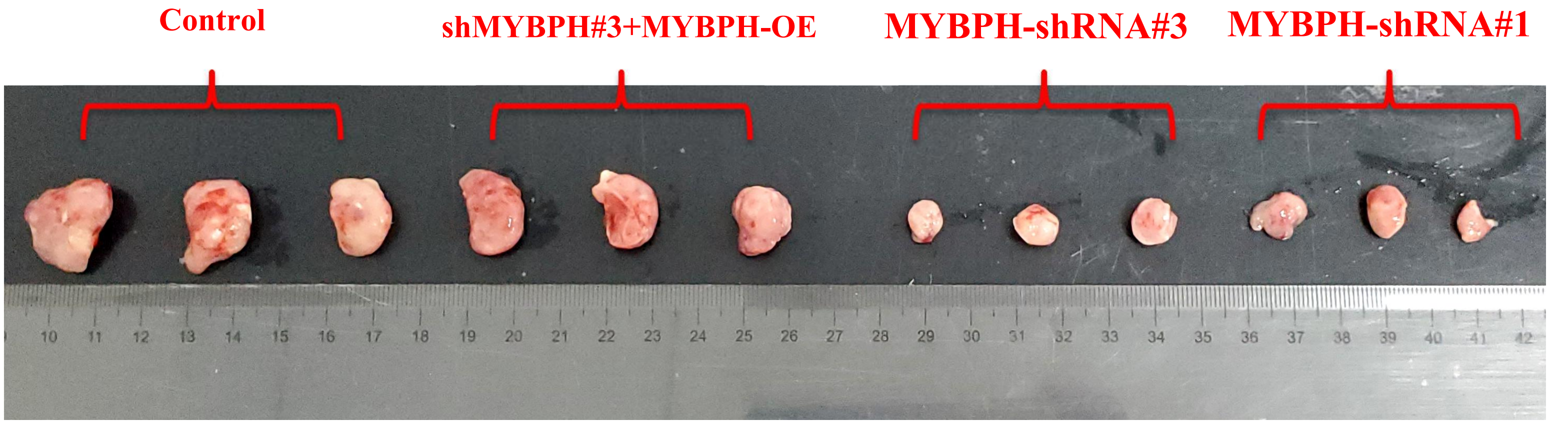

Figure S5. Mice xenograft models via the subcutaneous injection of U87 cells transfected with either shMYBPH (MYBPH-shRNA#1 and #3), shCtrl or shMYBPH#3+MYBPH-OE. At 48 h after transfection, U87 cells were injected into the left upper back of mice and allowed to grow until tumors formed.
